# Long-term gastrointestinal symptoms and sleep quality sequelae in adolescents after COVID-19: a retrospective study

**DOI:** 10.3389/fpubh.2024.1323820

**Published:** 2024-05-21

**Authors:** Wei-Lin Yang, Qi Wang, Ying Wang, Shaopeng Sun, Yan Shen, Lei-Min Yu

**Affiliations:** ^1^Department of Gastroenterology, Hangzhou TCM Hospital Affiliated to Zhejiang Chinese Medical University, Zhejiang Province, China; ^2^Department of Gastroenterology, Anji County Hospital of TCM, Huzhou, Zhejiang, China; ^3^Department of Neurology, Hangzhou TCM Hospital of Zhejiang Chinese Medical University (Hangzhou Hospital of Traditional Chinese Medicine), Hangzhou, Zhejiang, China; ^4^Key Laboratory of Digestive Pathophysiology of Zhejiang Province, The First School of Clinical Medicine, Zhejiang Chinese Medical University, Hangzhou, Zhejiang, China; ^5^Department of Gastroenterology, The Second Affiliated Hospital of Zhejiang Chinese Medical University (Xinhua Hospital of Zhejiang Province), Hangzhou, Zhejiang, China

**Keywords:** adolescents, COVID-19, long-term, gastrointestinal symptoms, sleep quality

## Abstract

**Objective:**

To evaluate the long-term gastrointestinal (GI) symptoms and sleep quality sequelae in adolescents with COVID-19.

**Methods:**

Between June and July 2023, an online survey was done in Xiaoshan District, Hangzhou City, Zhejiang Province, China, using the GI Symptom Rating Scale (GSRS) and the Pittsburgh Sleep Quality Inventory (PSQI).

**Results:**

GI symptoms in COVID-19 patients increased by 11.86% compared to before infection, while sleep quality decreased by 10.9%. Over time, there was a significant increase in the cumulative incidence rate of GI symptoms and sleep disorders (*p* < 0.001). Follow-up of COVID-19 positive patients within 6 months of infection showed that GI symptoms and sleep quality began to ease starting from the first month after infection. Further analysis indicated a significant linear relationship between the severity of GI symptoms and sleep quality (R > 0.5, *p* < 0.001). Moreover, females, older age, and higher education were identified as risk factors influencing the long-term effects of COVID-19.

**Conclusion:**

SARS-CoV-2 affects GI symptoms and sleep quality in adolescents during both the acute phase and post-infection periods. Over time, these symptoms gradually alleviate. A significant correlation exists between GI symptoms and sleep quality.

## 1 Introduction

Since SARS-CoV-2 emergence in Wuhan, China, in November 2019, it has rapidly spread worldwide, leading to its classification as a pandemic by the World Health Organization on March 11, 2020. There have been over 760 million confirmed cases of COVID-19 worldwide as of April 2023 (1). Apart from infecting the lungs, SARS-CoV-2 can also affect most organs. After recovery from SARS-CoV-2 infection, COVID-19 may be accompanied by long-term clinical sequelae, known as “Long-COVID” (2). It is a condition following SARS-CoV-2 infection characterized by persistent or recurring symptoms affecting multiple organs. The World Health Organization (WHO) defined Post COVID-19 Condition (PCC) in children as occurring in individuals with a history of confirmed or probable SARS-CoV-2 infection, with symptoms lasting at least two months that initially appeared within three months of acute COVID-19 (3). The National Institutes of Health (NIH) defined PASC as new, ongoing, or relapsing symptoms or conditions present 30 or more days after the diagnosis of SARS-CoV-2 infection or the onset of symptoms of COVID-19 (4).

During the pandemic, we have gained a better understanding of gastrointestinal (GI) symptoms following Covid-19 infection. The angiotensin-converting enzyme 2 (ACE2) receptors are expressed in the GI tract, allowing SARS-CoV-2 to enter the GI system through ACE2 receptor (5). Elmunzer BJ et al. (6) conducted a follow-up survey of patients 12–18 months after hospitalization for COVID-19, and found a high prevalence of DGBIs and persistent GI symptoms. Sleep is a crucial determinant of overall health and a vital component of normal adolescent development, particularly significant for students’ well-being and academic performance. Research findings indicated that COVID-19 patients frequently experienced poor sleep quality (7). Sleep problems have numerous negative effects on teenagers.

The bidirectional gut-brain interactions regulate essential physiological and homeostatic functions, and GI symptoms and sleep quality are related to these interactions. In December 2022, the most severe SARS-CoV-2 outbreak in history occurred in Zhejiang Province, China. Based on this event, we hypothesized that COVID-19 might affect GI symptoms and sleep patterns in adolescents. To investigate this, we conducted an online survey to elucidate the impact of COVID-19 on GI symptoms and sleep quality in adolescents, as well as to determine if there was a correlation between the two symptoms. Through this study, we aim to understand the persistent changes in GI symptoms and sleep quality after COVID-19, shedding light on the long-term adverse effects of the virus.

## 2 Methods

### 2.1 Participant information

From June to July 2020, we used a WeChat survey to recruit adolescent participants online in the Xiaoshan District of Hangzhou City, Zhejiang Province, aged between 12 and 17. All participants in the study responded to the survey by scanning a QR code on their mobile phones via WeChat. The questionnaire included all the items to be investigated and was presented in a single-choice format. We collected data from participants before infection, during the acute phase of infection, and at the 1st, 3rd, and 6th month follow-up periods after infection. The diagnosis of COVID-19 was based on the detection of SARS-CoV-2 positive by nasopharyngeal swab polymerase chain reaction or antigen positivity. Long COVID was defined as new, ongoing, or relapsing symptoms or conditions present 30 or more days after the diagnosis of SARS-CoV-2 infection or the onset of COVID-19 symptoms. PCC was defined for individuals with a history of confirmed or probable SARS-CoV-2 infection, where symptoms last at least two months and initially occur within three months of acute COVID-19.

Inclusion criteria Middle school and high school students residing in Xiaoshan District, Hangzhou City, who were willing to participate in the study and responded accurately to the questionnaire items were included.

Exclusion criteria Respondents whose questionnaire information was incomplete, failed to answer all questions, or contained contradictory information in the survey were excluded.

All participants (their parents or legal guardians in the case of children under 18) were informed of the research objectives and were assured of the confidentiality of their personal information. After obtaining their informed consent, the survey questionnaire was completed. The ethical approval institution is Medical Ethics Committee of Anji County Hospital of TCM (2023-038).

### 2.2 Questionnaire content

The survey questionnaire consisted of three sections.

1. Demographic and Medical History

This included age, gender, education level, previous GI symptoms, COVID-19 infection status, and hospitalization due to COVID-19.

1. GI Symptom Rating Scale (GSRS)

We surveyed participants using the GSRS for GI symptoms. It has 15 items rated on a 7-point scale, with a higher total score indicating more severe symptoms.

1. Pittsburgh Sleep Quality Inventory (PSQI)

PSQI assesses sleep quality on a scale of 0–21. Scores of 0–5 indicate very good sleep, 6–10 is fairly good, 11–15 is fairly bad, and 16–21 is very bad.

### 2.3 Statistical analysis

Data were sourced from WeChat questionnaires. Continuous variables were presented as mean ± standard deviation, and categorical ones as counts and percentages. Normal distribution categorical data was analyzed with the $\chi^{2}$ test, and continuous data with one-way ANOVA. Skewed data employed non-parametric rank sum tests. Paired data used paired $\chi^{2}$ and non-parametric rank sum tests for event rates and trends. Variable correlation was determined through the Spearman correlation test. A *p*-value of <0.05 indicated statistical significance. All analyses used SPSS version 26.0 (IBM, NY, USA).

## 3 Results

A total of 587 questionnaires were distributed, and all 587 were returned. After excluding 9 improperly filled or invalid questionnaires,578 valid questionnaires were obtained. Among them, there were 287 males and 291 females, with an average age of 15.087 ± 1.910 years. The COVID-19 infection rate was 91.700%. The incidence rate for PACS is 12.78%, and for PCC, it is 9.54%.

### 3.1 Pre-COVID-19 GI symptoms of respondents

Upon analyzing the overall symptoms of the GI tract, the research found that females were more likely than males to have a history of GI diseases (*p* < 0.05). Students with a history of GI diseases tended to be older than those without such history (*p* < 0.001). Moreover, there was a difference in the distribution of education levels among those with a history of GI diseases (*p* < 0.001). Specifically, 10th and 11th-grade students were more likely to have a history of GI diseases compared to those in the 6th, 7th, and 8th grades (*p* < 0.002) (Table 1).

**Table 1:** Baseline gastrointestinal symptoms fact sheet.

	Gastrointestinal symptoms		Statistic	*p*
	No	Yes		
**Gender**				
Male	79	208	$\chi^{2}$ = 218.926	<0.001
Female	64	227		
Age (years)	14.67 ± 1.891	15.38 ± 1.872	*F* = 19.217	<0.001
**Education level**				
6th grade	31	29	$\chi^{2}$ = 34.815	<0.001
7th grade	73	62		
8th grade	56	61		
9th grade	24	30		
10th grade	17	47		
11th grade	20	63		
12th grade	17	45		
**Gastrointestinal history**				
No	227	262	$\chi^{2}$ = 33.684	<0.001
Yes	12	77		

Furthermore, after analyzing the 15 sets of GI symptoms on the GSRS scale, it was found that, except for the four groups of symptoms: acid reflux, heartburn, loose stools, and nausea, the remaining 11 GI symptoms were significantly related to gender. Specifically, girls were more prone to experiencing these discomfort symptoms (*p* < 0.05). Additionally, age and educational level were significantly associated with all 15 GI symptoms (*p* < 0.05). Students who exhibited various GI symptoms were often in higher grades. Compared to sixth-grade students and middle school students, high school students were more likely to experience four or more GI symptoms (*p* = 2.1354e-11).

### 3.2 Pre-COVID-19 sleep quality of respondents

The study found that gender was correlated with sleep quality (*p* = 0.025), indicating that males had better sleep quality than females before infection. Age also showed a differential distribution among different sleep quality groups (*p* = 0.000001). Students with “very good” sleep quality differed in age from those whose sleep quality was “fairly good” (*p* = 0.000004). These results indicated that older students were more prone to poor sleep quality. Educational level was associated with sleep quality (*p* = 4.3387e-10). Compared to sixth graders and middle school students, high school students demonstrated poorer sleep quality (*p* < 0.001) (Table 2).

**Table 2:** Baseline sleep quality fact sheet.

	Very good	Fairly good	Fairly bad	Very bad	Statistic	*p*
**Gender**						
Male	222	54	7	2	Z = −2.236	0.025
Female	202	67	16	3		
Age (years)	14.83 ± 1.894	15.81 ± 1.796	16.04 ± 1.665	15.20 ± 2.168	*F* = 10.466	<0.001
**Education level**						
6th grade	50	7	2	1	H (K) = 55.142	<0.001
7th grade	116	16	1	1		
8th grade	96	17	2	0		
9th grade	42	9	2	0		
10th grade	40	19	3	1		
11th grade	45	29	8	1		
12th grade	33	23	5	1		
**Gastrointestinal history**						
No	373	94	13	5	Z = −3.885	<0.001
Yes	51	27	10	0		

### 3.3 GI symptom changes post-COVID-19

**Figure 1:**
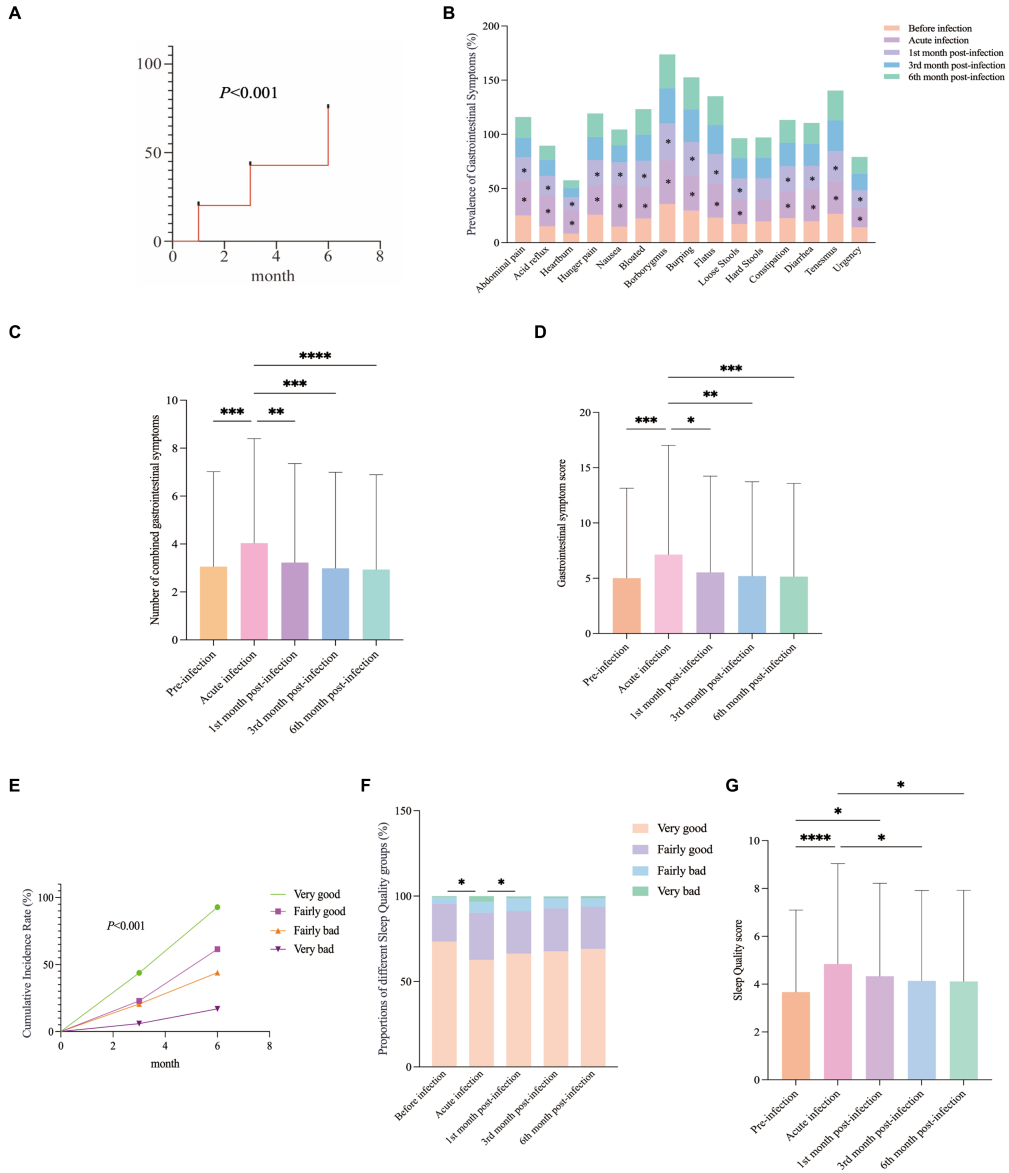
GI Symptoms and Sleep Quality Changes Post-COVID-19: **(A)** Cumulative incidence of GI symptoms. **(B)** During the acute phase of COVID-19, the incidence rates of 15 GI symptoms (excluding “hard stools”) rose significantly. Post-infection, these rates consistently improved, with the most notable improvement in the 1st month. **(C,D)** The combined number of GI symptoms and the GI symptom score varied at different follow-up times. **(E)** Overall distribution of sleep quality scores during the follow-up period. **(F)** The distribution of sleep quality at different follow-up time points. **(G)** Sleep Quality ratings at various follow-up time points.

During the COVID-19 pandemic, GI symptoms in COVID-19 patients rose by 11.86% compared to before infection. After COVID-19 infection, respondents were followed up at 1, 3, and 6 months. A gradual increase in GI symptom incidence was observed over time (Figure 1A). During the acute phase of COVID-19, the incidence rates of 15 GI symptoms (excluding “hard stools”) rose significantly (Figure 1B). This trend was also reflected in the combined number of GI symptoms and the severity scores (Figures 1C,D). Our study revealed an exacerbation during the acute phase of the COVID-19 infection, followed by improvement in the first month post-infection. By the 3rd and 6th months post-infection, the GI symptoms of the participants had increased compared to before the infection, but the increase was not statistically significant (*p* > 0.05) (Figures 1C,D). This result suggested that GI symptoms had gradually decreased over time.

### 3.4 Sleep quality changes post-COVID-19

Our research found that the sleep quality of the surveyed population decreased by 10.9% after being infected with the novel coronavirus. compared to the period before the COVID-19 pandemic, there was a trend of deteriorating sleep quality among infected individuals during the acute infection phase (*p* = 3.4782e-7). Specifically, the sleep quality of respondents deteriorated after acute infection, with an increased proportion of them reporting sleep quality as “fairly good,” “fairly bad,” and “very bad” (Table 3).

**Table 3:** Sleep quality during acute COVID-19.

	Before infection				Statistic	*p*
	Very good	Fairly good	Fairly bad	Very bad		
**Acute infection**						
Very good	341 [180,161]	23 [7,16]	2 [0,2]	0 [0,0]	$\chi^{2}$ = 309.573	<0.001
Fairly good	63 [34,29]	79 [37,42]	4 [2,2]	1 [1,0]		
Fairly bad	9 [6,3]	13 [8,5]	12 [4,8]	1 [0,1]		
Very bad	8 [2,6]	4 [2,2]	4 [1,3]	2 [0,2]		

The cumulative incidence of different sleep quality categories gradually increased during the follow-up period (Figure 1E). During the acute phase of COVID-19 infection, the proportion of individuals reporting “good sleep” quality decreased, while the proportions reporting “fairly good,” “fairly bad” and “very bad” sleep quality increased significantly (Figure 1F). Starting from the first month after infection, the sleep quality of the participants had begun to gradually improve. Follow-up data from the 3rd and 6th months after infection had shown that their sleep quality had still been worse than before the infection, but the difference was not statistically significant (*p* > 0.05) (Figure 1G). This finding suggested that sleep quality would have gradually recovered over time.

### 3.5 Correlation between GI symptoms and sleep quality

**Figure 2:**
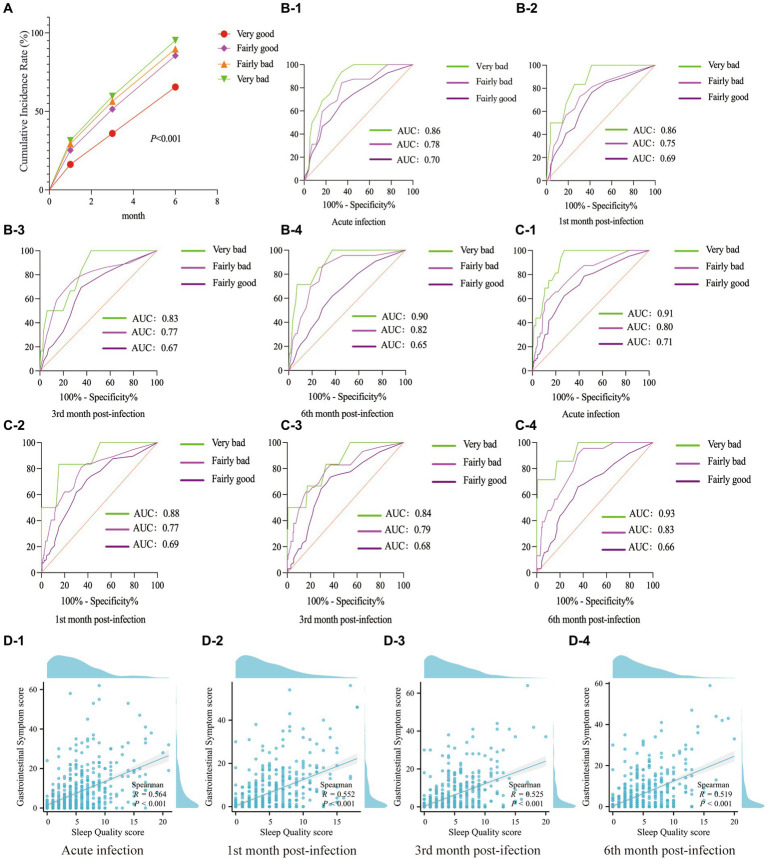
The correlation between GI symptoms and sleep quality. **(A)** Cumulative incidence rate of GI symptoms. **(B)** ROC curves related sleep quality to GI symptom count up to 6 months post-acute COVID-19. **(C)** During the follow-up period, ROC curves were constructed to assess the relationship between sleep quality categories and the severity of GI symptoms. **(D)** Scatter plots showed the correlation between sleep quality scores and the severity of GI symptoms during the acute phase of COVID-19 infection, as well as at the 1, 3, and 6-month follow-ups.

Different categories of sleep quality led to varied cumulative incidence rates of GI symptoms: The category with “very bad” sleep quality was the most noticeable, while the “very good” sleep quality category was not significant (Figure 2A). Using the GI symptoms related to “very good” sleep quality as a reference, statistical analysis suggested that compared to “very good” sleep quality, “very bad” sleep quality had the strongest specificity with the severity of GI symptoms, followed by “fairly bad” sleep quality (Figures 2B,C). This study found that among COVID-19 patients during the acute infection phase and the subsequent 1, 3, and 6-month follow-up periods, there was a correlation between sleep quality scores and the severity of GI symptoms (R > 0.5, *p* < 0.001) (Figure 2D).

### 3.6 Risk factors for long COVID-19 with GI symptoms

As previously mentioned, following the outbreak of the COVID-19 pandemic, there was an increase in the prevalence of GI symptoms among infected individuals. Notably, during the acute phase of COVID-19, there was a significant rise in the occurrence of GI symptoms among females (OR = 33.750, 95% CI: 13.631–83.563, *p* = 3.2437e-8) and older age groups of students (*p* = 0.000566).

GI symptoms in COVID-19-infected individuals started improving as early as the first month after infection, with males showing the most significant improvement (*p* = 0.000002). By the end of the follow-up, compared to the acute infection phase and the first month after infection, all COVID-19 patients’ GI symptoms had significantly improved, and there was no significant difference between genders (*p* > 0.05). We also observed that the student population experiencing GI symptoms tended to be older in age (*p* < 0.01), and this phenomenon persisted even as the follow-up period extended. During the first month following COVID-19 infection, there was a notable improvement in GI symptoms among sixth-grade and middle school students (*p* < 0.01). However, in the subsequent follow-up period, there were no discernible differences based on education level (*p* > 0.05). Additional data are described in Supplementary Table S12.

### 3.7 Risk factors for long COVID-19 with sleep quality

Conducted follow-up assessments at the 1st month, 3rd month, and 6th month after COVID-19 infection, we stratified the data by gender, age, and educational level. The study showed that female infected individuals exhibited lower sleep quality in comparison (*p* = 3.978e-9). The sleep quality of COVID-19-infected individuals began to improve as early as the first month post-infection, with males showing the most noticeable improvement (*p* = 0.007453). By the end of the follow-up period, compared to the acute infection phase (*p* = 0.005298) and the first month after infection (*p* = 0.016940), the sleep quality of male COVID-19 patients had shown a more significant improvement. In the first month following COVID-19 infection, there was a noticeable improvement in the sleep quality of middle school students (*p* < 0.01). However, in the subsequent follow-up period, there were no differences based on education level (*p* > 0.05). Additional data are described in Supplementary Table S13.

## 4 Discussion

Adolescents are the future pillars of a nation, playing a crucial role in societal advancement. This transformative phase is sensitive, making them susceptible to particular physical and psychological challenges. The outbreak of COVID-19 garnered significant global attention. Given that the coronavirus can adversely affect the GI tract and sleep quality, we conducted a retrospective study on GI symptoms and sleep quality in adolescents in a local area within six months following a coronavirus infection. The purpose of this study was to explore the long-term effects of COVID-19 on GI symptoms and sleep quality in adolescents. Our research indicated that GI symptoms might have persisted after SARS-CoV-2 infection, and that contracting SARS-CoV-2 could have led to a decline in sleep quality. There was a correlation between GI symptoms and sleep quality. However, these symptoms gradually improved with time. Being female, and older in age were associated risk factors. Consistent with other studies (8, 9).

After a SARS-CoV-2 infection, acute and chronic GI symptoms are common. In children, GI symptoms caused by the coronavirus infection can be as high as 84.1% (10). The incidence of irritable bowel syndrome (IBS) in children and adolescents increased from 3.8% before the pandemic to 8.8% during the pandemic (11). Weng J et al.’s study found that 90 days post-infection, the most common GI sequelae were loss of appetite (24%), nausea (18%), acid reflux (14%), and diarrhea (15%) among patients (12). Our research showed that GI symptoms in COVID-19 patients rose by 11.86%, and these symptoms persisted after SARS-CoV-2 infection. Interestingly, we found that GI symptoms were most severe during the acute phase of the infection, and the symptoms began to improve in the first month of follow-up. Liver injuries occur in 14–53% of COVID-19 cases (13). Cooper S. et al. (14) reported that in children with COVID-19, possible long-term liver complications include acute liver failure and hepatitis with cholestasis, noting that unlike adult cohorts, the pediatric cohort did not exhibit steatosis. Ashktorab H et al. (15) found that Long-COVID patients with abnormal liver enzymes, a history of liver disease, or positive Hepatitis surface antigen have a higher risk of sleep problems. We will conduct relevant research in the future.

The reasons for GI symptoms caused by the SARS-CoV-2 are multifaceted. Through sgN mRNA, Ribeiro IP et al. (16) detected replicating SARS-CoV-2 in RS samples, suggesting the possibility of fecal-oral transmission of the virus. Additionally, researchers found evidence of SARS-CoV-2 shedding in feces persisting for up to 2 months (17). This information further provides evidence of the virus’s replication and continuous presence in the GI tract. SARS-CoV-2 targeted GI cells that expressed ACE2, leading to inflammatory responses and the release of cytokines (18), thereby impairing the gut barrier function (19). Research had shown that up to 6 months after acute infection, the presence of SARS-CoV-2 nucleic acid was found in the small intestines of COVID-19 survivors, accompanied by persistent immune activation, hypothesized as the cause of persistent GI symptoms post-COVID-19 (20). Moreover, the gut microbiota of the COVID-19 patients underwent changes (21). Dysbiosis of the patient’s gut microbiota modulated the host immune response, leading to long-term GI symptoms (22).

In our study, we found that being female was a risk factor for persistent GI symptoms after infection with the novel coronavirus. Mehreen Siyal and colleagues (18) conducted a prospective follow-up of 300 hospitalized COVID-19 patients without a history of IBS after their discharge and found an increased risk of IBS in female patients post-COVID-19. In a meta-analysis conducted by Korterink et al. (23), the proportion of girls with functional abdominal pain was significantly higher than that of boys. Sex hormones might have been the reason behind this gender difference, as they played roles in influencing the gut-brain axis, stress responses, visceral sensitivity and motility, intestinal barrier functions, mucosal immune activation, and the gut microbiome (24).

We further focused on sleep conditions associated with functional GI diseases. COVID-19 significantly disrupted sleep patterns, leading to a general rise in sleep disturbances (25). Adequate sleep is crucial for physical and mental health, playing a significant role especially in emotional regulation, cognition, psychosocial development, and physical growth (26). Hoang HTX et al. conducted a cross-sectional online survey of 1,056 non-hospitalized COVID-19 survivors within six months of their initial infection and found that 76.1% had insomnia, with 22.8% of them experiencing severe insomnia (27). Our research showed that infection with SARS-CoV-2 led to a decline in sleep quality, and this symptom persisted. Similarly, we found that the decline in sleep quality was most severe during the acute phase of the infection, but symptoms began to improve in the first month of follow-up.

The reasons for the decline in sleep quality among adolescents after contracting the coronavirus are multifaceted. Lockdown measures during the COVID-19 pandemic, such as stressful events, prolonged confinement, school closures, and increased online activity, have impacted adolescent mental health (26, 28), potentially affecting their sleep quality. In a survey of 2,291 Italians, 57.1% reported poor sleep quality during the COVID-19 pandemic, and there was a significant correlation between symptoms of sleep quality, generalized anxiety, psychological distress, and COVID-19-related Post-Traumatic Stress Disorder (PTSD) symptoms (29). Additionally, the coronavirus itself has the characteristic of invading brain tissue. Paniz-Mondolfi A et al. (30) analyzed brains of COVID-19 victims and found the virus in neurons and endothelial cells of the frontal lobe. Recently, SARS-CoV-2 was thought to affect the central nervous system (CNS) by interacting with the blood–brain barrier (BBB), and it was observed that SARS-CoV-2 infection disrupted the BBB through Wnt signaling (31). Therefore, the sleep quality of adolescents is shaped by a myriad of factors, including sociopsychological stressors and the impact of the COVID-19 virus.

In our study, we found after the infection of SARS-CoV-2, female patients exhibited a significant decline in sleep quality. Tański W et al. (32) aimed to evaluate the impact of past COVID-19 on sleep disturbances and noted that these disturbances, lasting at least one month, began after recovery and that female gender significantly predicted the severity of insomnia. The previous research suggested that estradiol mediates sleep gender differences by acting on neurons in the ventrolateral preoptic area, leading to earlier onset of slow-wave sleep in adolescent girls, which subsequently delayed the circadian rhythm and resulted in objective sleep disturbances (33).

The interactions within the gut-brain axis are thought to be vital in regulating sleep, chronic abdominal pain, and GI functions (34). In our study, we found a correlation between GI symptoms and sleep quality among adolescents during the backdrop of the COVID-19 pandemic, suggesting a potential link to gut-brain axis dysregulation. Other studies similarly suggested that, compared to their peers, children and adolescents with IBS had inferior sleep quality (35), while students with had poor sleep were more likely to develop IBS than those without IBS (36). Additionally, our research found that the population with GI symptoms and decreased sleep quality tended to be older and had a higher level of education, which we speculated might be related to the particularity of Chinese education. Older students, facing the pressures of advancement exams, experience sleep disturbances such as late bedtimes, early awakenings, irregular sleep patterns, and sleep deficiencies. This can, in turn, disrupt the gut-brain axis function, leading to GI symptoms and a decline in sleep quality.

Our study has several limitations. First, it might have selection bias due to the survey’s timing, which overlapped with China’s academic examination season. This likely led to reduced participation, especially among 6th, 9th, and 12th-grade students, who were preparing for exams and undergoing educational transitions, thereby affecting the diversity and representativeness of the sample. Second, using self-reported symptoms as data sources may introduce inaccuracies. Furthermore, the small sample size limits the robustness and generalizability of our findings. Future research should aim to overcome these limitations by including a larger and more diverse sample to better represent the population, as well as implementing a longer follow-up period. Furthermore, including other variables, such as mood and body mass index (BMI), will provide a more comprehensive view of factors influencing the study’s results and aid in developing clinical prediction models. Additionally, we plan to conduct a new round of investigation in the future to clarify the difference in incidence rates between PACS and PCC.

In summary, after a 6-month follow-up period, COVID-19 infections caused persistent changes in adolescents’ GI symptoms and sleep quality; however, these symptoms gradually improved over time. Declines in gastrointestinal symptoms and sleep quality in adolescents can lead to numerous societal and health issues. Importantly, our study found a potential link between gastrointestinal symptoms and sleep problems in Long-COVID patients. Our results contribute to the evidence on the potential long-term effects of COVID-19 on multiple organ systems, including the gastrointestinal and nervous systems. We recommend that schools organize workshops on mental and physical regulation, coordinated by psychologists and gastroenterologists, to provide adolescents with guidance on improving gastrointestinal symptoms and sleep quality.

## Data availability statement

The original contributions presented in the study are included in the article/Supplementary material, further inquiries can be directed to the corresponding author.

## Ethics statement

The studies involving humans were approved by Medical Ethics Committee of Anji County Hospital of TCM (2023-038). The studies were conducted in accordance with the local legislation and institutional requirements. Written informed consent for participation in this study was provided by the participants’ legal guardians/next of kin.

## Author contributions

W-LY: Writing – original draft, Conceptualization, Investigation, Methodology. QW: Investigation, Project administration, Supervision, Writing – review & editing. YW: Writing – review & editing, Data curation. SS: Data curation, Writing – review & editing, Conceptualization. YS: Writing – review & editing, Funding acquisition. L-MY: Conceptualization, Investigation, Methodology, Writing – original draft.
