# Supplementary material for: Long-term gastrointestinal symptoms and sleep quality sequelae in adolescents after COVID-19: a retrospective study
Source: Front Public Health. 2024 May 21;12:1323820. doi: 10.3389/fpubh.2024.1323820 (PMC11148350; doi:10.3389/fpubh.2024.1323820)
Supplement: Supplementary file 1 [file Table_1.DOCX]

**Supplementary material**

**Table 1 Correlation Between 15 Gastrointestinal Symptoms and Educational Level (Before Infection)**

|  | **Abdominal pain** | | **Acid reflux** | | **Heartburn** | | **Hunger pain** | | **Nausea** | | **Bloated** | | **Borborygmus** | | **Burping** | | **Flatus** | | **Loose Stools** | | **Hard Stools** | | **Constipation** | | **Diarrhea** | | **Tenesmus** | | **Urgency** | |
| --- | --- | --- | --- | --- | --- | --- | --- | --- | --- | --- | --- | --- | --- | --- | --- | --- | --- | --- | --- | --- | --- | --- | --- | --- | --- | --- | --- | --- | --- | --- |
|  | No | Yes | No | Yes | No | Yes | No | Yes | No | Yes | No | Yes | No | Yes | No | Yes | No | Yes | No | Yes | No | Yes | No | Yes | No | Yes | No | Yes | No | Yes |
| **6^th^ grade** | 52 | 8 | 58 | 2 | 60 | 0 | 53 | 7 | 55 | 5 | 56 | 4 | 46 | 14 | 49 | 11 | 49 | 11 | 55 | 5 | 53 | 7 | 56 | 4 | 55 | 5 | 51 | 9 | 52 | 8 |
| **7^th^ grade** | 115 | 20 | 120 | 15 | 131 | 4 | 109 | 26 | 120 | 15 | 119 | 16 | 101 | 34 | 106 | 29 | 119 | 16 | 125 | 10 | 119 | 16 | 114 | 21 | 121 | 14 | 117 | 18 | 123 | 12 |
| **8^th^ grade** | 95 | 22 | 107 | 10 | 114 | 3 | 91 | 26 | 106 | 11 | 98 | 19 | 88 | 29 | 95 | 22 | 96 | 21 | 103 | 14 | 102 | 15 | 100 | 17 | 102 | 15 | 102 | 15 | 107 | 10 |
| **9^th^ grade** | 41 | 13 | 50 | 4 | 48 | 6 | 45 | 9 | 51 | 3 | 46 | 8 | 42 | 12 | 47 | 7 | 48 | 6 | 48 | 6 | 49 | 5 | 46 | 8 | 48 | 6 | 44 | 10 | 49 | 5 |
| **10^th^ grade** | 41 | 23 | 46 | 18 | 51 | 13 | 39 | 25 | 50 | 14 | 47 | 17 | 36 | 28 | 41 | 23 | 45 | 19 | 52 | 12 | 50 | 14 | 50 | 14 | 46 | 18 | 42 | 22 | 50 | 14 |
| **11^th^ grade** | 56 | 27 | 66 | 17 | 75 | 8 | 53 | 30 | 66 | 17 | 46 | 37 | 32 | 51 | 38 | 45 | 51 | 32 | 57 | 26 | 52 | 31 | 49 | 34 | 54 | 29 | 39 | 44 | 65 | 18 |
| **12^th^ grade** | 40 | 22 | 46 | 16 | 51 | 11 | 40 | 22 | 47 | 15 | 39 | 23 | 34 | 28 | 36 | 26 | 39 | 23 | 41 | 21 | 41 | 21 | 38 | 24 | 41 | 21 | 35 | 27 | 50 | 12 |
| **Statistic** | $\chi^{2}$=24.800 | | $\chi^{2}$=31.601 | | $\chi^{2}$=37.480 | | $\chi^{2}$=26.260 | | $\chi^{2}$=19.430 | | $\chi^{2}$=54.595 | | $\chi^{2}$=49.433 | | $\chi^{2}$=52.432 | | $\chi^{2}$=52.432 | | $\chi^{2}$=41.241 | | $\chi^{2}$=40.292 | | $\chi^{2}$=45.370 | | $\chi^{2}$=42.485 | | $\chi^{2}$=72.147 | | $\chi^{2}$=15.912 | |
| ***P*** | 0.000372 | | 0.000003 | | 3.6638e-7 | | 0.000199 | | 0.000268 | | 7.3825e-12 | | 6.565e-9 | | 5.5943e-9 | | 0.000002 | | 2.5959e-7 | | 3.9907e-7 | | 9.464e-10 | | 1.1402e-9 | | 3.8865e-14 | | 0.002078 | |

^a^ The chi-square test was employed to examine the correlation between variables, with a significance level set at *P*<0.05 indicating statistical significance.

^b^ The results revealed a statistically significant correlation between all 15 gastrointestinal symptoms and educational level.

**Table 2 Correlation Between 15 Gastrointestinal Symptoms and Educational Level (Acute Infection)**

|  | **Acute infection** | | | | | | | | | | | | | | | | | | | | | | | | | | | | | |
| --- | --- | --- | --- | --- | --- | --- | --- | --- | --- | --- | --- | --- | --- | --- | --- | --- | --- | --- | --- | --- | --- | --- | --- | --- | --- | --- | --- | --- | --- | --- |
|  | **Abdominal pain** | | **Acid reflux** | | **Heartburn** | | **Hunger pain** | | **Nausea** | | **Bloated** | | **Borborygmus** | | **Burping** | | **Flatus** | | **Loose Stools** | | **Hard Stools** | | **Constipation** | | **Diarrhea** | | **Tenesmus** | | **Urgency** | |
|  | No | Yes | No | Yes | No | Yes | No | Yes | No | Yes | No | Yes | No | Yes | No | Yes | No | Yes | No | Yes | No | Yes | No | Yes | No | Yes | No | Yes | No | Yes |
| **Before infection** |  |  |  |  |  |  |  |  |  |  |  |  |  |  |  |  |  |  |  |  |  |  |  |  |  |  |  |  |  |  |
| **6^th^ grade** |  |  |  |  |  |  |  |  |  |  |  |  |  |  |  |  |  |  |  |  |  |  |  |  |  |  |  |  |  |  |
| No | 39 | 10 | 46 | 9 | 50 | 7 | 45 | 7 | 35 | 17 | 43 | 11 | 37 | 8 | 41 | 7 | 37 | 9 | 47 | 5 | 45 | 5 | 50 | 4 | 44 | 8 | 45 | 5 | 47 | 3 |
| Yes | 2 | 6 | 1 | 1 | 0 | 0 | 2 | 3 | 1 | 4 | 1 | 2 | 3 | 9 | 2 | 7 | 0 | 11 | 0 | 5 | 3 | 4 | 1 | 2 | 0 | 5 | 1 | 6 | 0 | 7 |
| **7^th^ grade** |  |  |  |  |  |  |  |  |  |  |  |  |  |  |  |  |  |  |  |  |  |  |  |  |  |  |  |  |  |  |
| No | 85 | 18 | 91 | 17 | 105 | 13 | 88 | 10 | 76 | 31 | 100 | 9 | 82 | 9 | 88 | 9 | 98 | 10 | 108 | 5 | 107 | 2 | 99 | 4 | 94 | 14 | 98 | 6 | 104 | 6 |
| Yes | 9 | 10 | 4 | 10 | 1 | 3 | 6 | 18 | 3 | 12 | 4 | 9 | 7 | 24 | 9 | 16 | 2 | 12 | 3 | 6 | 4 | 9 | 3 | 16 | 3 | 11 | 2 | 16 | 1 | 11 |
| **8^th^ grade** |  |  |  |  |  |  |  |  |  |  |  |  |  |  |  |  |  |  |  |  |  |  |  |  |  |  |  |  |  |  |
| No | 64 | 14 | 75 | 14 | 82 | 14 | 69 | 6 | 69 | 19 | 70 | 10 | 59 | 11 | 69 | 8 | 65 | 13 | 78 | 8 | 80 | 5 | 77 | 5 | 74 | 11 | 78 | 6 | 84 | 6 |
| Yes | 5 | 16 | 2 | 8 | 0 | 3 | 3 | 21 | 3 | 8 | 4 | 15 | 5 | 24 | 4 | 18 | 4 | 17 | 1 | 12 | 1 | 13 | 1 | 16 | 1 | 13 | 2 | 13 | 1 | 8 |
| **9^th^ grade** |  |  |  |  |  |  |  |  |  |  |  |  |  |  |  |  |  |  |  |  |  |  |  |  |  |  |  |  |  |  |
| No | 28 | 10 | 33 | 14 | 35 | 10 | 39 | 3 | 34 | 14 | 36 | 7 | 32 | 7 | 38 | 6 | 38 | 7 | 39 | 6 | 44 | 2 | 36 | 7 | 39 | 6 | 38 | 3 | 44 | 2 |
| Yes | 2 | 11 | 1 | 3 | 0 | 6 | 3 | 6 | 0 | 3 | 2 | 6 | 3 | 9 | 3 | 4 | 1 | 5 | 2 | 4 | 0 | 5 | 1 | 7 | 2 | 4 | 1 | 9 | 0 | 5 |
| **10^th^ grade** |  |  |  |  |  |  |  |  |  |  |  |  |  |  |  |  |  |  |  |  |  |  |  |  |  |  |  |  |  |  |
| No | 29 | 9 | 37 | 6 | 38 | 10 | 34 | 3 | 34 | 13 | 37 | 7 | 27 | 6 | 35 | 3 | 36 | 6 | 41 | 8 | 44 | 3 | 43 | 3 | 33 | 10 | 36 | 3 | 45 | 2 |
| Yes | 4 | 18 | 2 | 15 | 0 | 12 | 8 | 15 | 0 | 13 | 1 | 15 | 7 | 20 | 4 | 18 | 3 | 15 | 0 | 11 | 0 | 13 | 3 | 11 | 3 | 14 | 2 | 19 | 1 | 12 |
| **11^th^ grade** |  |  |  |  |  |  |  |  |  |  |  |  |  |  |  |  |  |  |  |  |  |  |  |  |  |  |  |  |  |  |
| No | 39 | 14 | 47 | 17 | 54 | 18 | 44 | 7 | 37 | 27 | 40 | 4 | 22 | 8 | 32 | 3 | 45 | 5 | 50 | 4 | 49 | 0 | 44 | 3 | 41 | 11 | 34 | 4 | 59 | 3 |
| Yes | 10 | 17 | 2 | 14 | 2 | 6 | 5 | 24 | 1 | 15 | 2 | 34 | 5 | 45 | 2 | 43 | 1 | 29 | 0 | 26 | 5 | 26 | 5 | 28 | 3 | 25 | 1 | 41 | 0 | 18 |
| **12^th^ grade** |  |  |  |  |  |  |  |  |  |  |  |  |  |  |  |  |  |  |  |  |  |  |  |  |  |  |  |  |  |  |
| No | 32 | 5 | 35 | 8 | 43 | 5 | 36 | 1 | 31 | 13 | 33 | 4 | 26 | 6 | 29 | 4 | 32 | 5 | 35 | 3 | 38 | 0 | 33 | 2 | 35 | 3 | 31 | 2 | 45 | 2 |
| Yes | 7 | 15 | 1 | 15 | 2 | 9 | 6 | 16 | 1 | 14 | 2 | 20 | 1 | 26 | 2 | 24 | 1 | 21 | 1 | 20 | 1 | 20 | 4 | 20 | 1 | 20 | 3 | 23 | 0 | 12 |
| **Statistic** | $\chi^{2}$=75.786 | | $\chi^{2}$=137.352 | | $\chi^{2}$=124.443 | | $\chi^{2}$=227.762 | | $\chi^{2}$=96.738 | | $\chi^{2}$=240.189 | | $\chi^{2}$=228.412 | | $\chi^{2}$=265.223 | | $\chi^{2}$=256.319 | | $\chi^{2}$=292.058 | | $\chi^{2}$=352.031 | | $\chi^{2}$=302.893 | | $\chi^{2}$=214.534 | | $\chi^{2}$=346.400 | | $\chi^{2}$=357.242 | |
| ***P*** | 0.000215 | | 3.9847e-14 | | 1.2046e-17 | | 0.720203 | | 1.0236e-29 | | 0.000014 | | 0.012673 | | 0.108857 | | 1.0273e-7 | | 0.000002 | | 0.720100 | | 0.183925 | | 5.0449e-9 | | 0.011508 | | 0.000049 | |

^a^ The analysis excluded individuals who were not infected with COVID-19, and a paired chi-square test was conducted to examine the correlation between variables, with a significance level set at *P*<0.05 indicating statistical significance.

^b^ The results indicated that, apart from hunger pain, burping, hard stools, and constipation, the incidence rates of all other gastrointestinal symptoms following COVID-19 infection were correlated with educational level.

**Table 3 Correlation Between 15 Gastrointestinal Symptoms and Gender (Before Infection)**

|  | **Male** | **Female** | **Statistic** | ***P*** |
| --- | --- | --- | --- | --- |
| **Abdominal pain** |  |  |  |  |
| Yes | 54 | 82 | $\chi^{2}$=7.041 | 0.008 |
| No | 233 | 209 |  |  |
| **Acid reflux** |  |  |  |  |
| Yes | 35 | 48 | $\chi^{2}$=2.172 | 0.141 |
| No | 252 | 243 |  |  |
| **Heartburn** |  |  |  |  |
| Yes | 17 | 28 | $\chi^{2}$=2.753 | 0.097 |
| No | 270 | 263 |  |  |
| **Hunger pain** |  |  |  |  |
| Yes | 53 | 92 | $\chi^{2}$=13.292 | <0.001 |
| No | 234 | 199 |  |  |
| **Nausea** |  |  |  |  |
| Yes | 35 | 46 | $\chi^{2}$=1.565 | 0.211 |
| No | 252 | 245 |  |  |
| **Bloated** |  |  |  |  |
| Yes | 42 | 83 | $\chi^{2}$=190.218 | <0.001 |
| No | 245 | 208 |  |  |
| **Borborygmus** |  |  |  |  |
| Yes | 83 | 114 | $\chi^{2}$=6.764 | 0.009 |
| No | 204 | 177 |  |  |
| **Burping** |  |  |  |  |
| Yes | 42 | 83 | $\chi^{2}$=104.685 | <0.001 |
| No | 245 | 208 |  |  |
| **Flatus** |  |  |  |  |
| Yes | 54 | 77 | $\chi^{2}$=5.014 | 0.025 |
| No | 235 | 214 |  |  |
| **Loose Stools** |  |  |  |  |
| Yes | 40 | 56 | $\chi^{2}$=3.065 | 0.080 |
| No | 249 | 235 |  |  |
| **Hard Stools** |  |  |  |  |
| Yes | 43 | 68 | $\chi^{2}$=6.752 | 0.009 |
| No | 246 | 223 |  |  |
| **Constipation** |  |  |  |  |
| Yes | 46 | 79 | $\chi^{2}$=103541 | 0.001 |
| No | 241 | 212 |  |  |
| **Diarrhea** |  |  |  |  |
| Yes | 44 | 64 | $\chi^{2}$=4.221 | 0.040 |
| No | 243 | 227 |  |  |
| **Tenesmus** |  |  |  |  |
| Yes | 62 | 85 | $\chi^{2}$=4.409 | 0.036 |
| No | 225 | 206 |  |  |
| **Urgency** |  |  |  |  |
| Yes | 30 | 49 | $\chi^{2}$=2.441 | 0.025 |
| No | 257 | 242 |  |  |

^a^ A paired chi-square test was conducted to examine the correlation between variables, with a significance level set at *P*<0.05 indicating statistical significance.

^b^ The results indicated that, apart from acid reflux, heartburn, loose stools, and nausea, the incidence rates of all other gastrointestinal symptoms before COVID-19 infection were correlated with gender.

**Table 4 Correlation Between 15 Gastrointestinal Symptoms and Gender (Acute Infection)**

|  | **Acute infection** | | **Statistic** | ***P*** |
| --- | --- | --- | --- | --- |
|  | **Yes** | **No** |  |  |
| **Abdominal pain** |  |  |  |  |
| Yes | 94(38,56) | 39(15,24) | $\chi^{2}$=115.330 | 0.000215 |
| No | 79(44,36) | 318(164,153) |  |  |
| **Acid reflux** |  |  |  |  |
| Yes | 67(28,39) | 13(6,7) | $\chi^{2}$=138.208 | 2.289e-14 |
| No | 86(43,43) | 364(184,180) |  |  |
| **Heartburn** |  |  |  |  |
| Yes | 39(14,25) | 5(2,3) | $\chi^{2}$=123.579 | 6.4039e-18 |
| No | 78(47,31) | 408(198,210) |  |  |
| **Hunger pain** |  |  |  |  |
| Yes | 104(34,70) | 33(16,17) | $\chi^{2}$=227.275 | 0.635308 |
| No | 38(26,12) | 355(185,170) |  |  |
| **Nausea** |  |  |  |  |
| Yes | 70(28,42) | 9(6,3) | $\chi^{2}$=95.574 | 5.4566e-30 |
| No | 135(69,66) | 316(158,158) |  |  |
| **Bloated** |  |  |  |  |
| Yes | 102(32,70) | 16(7,9) | $\chi^{2}$=242.486 | 0.000014 |
| No | 52(26,26) | 360(196,164) |  |  |
| **Borborygmus** |  |  |  |  |
| Yes | 158(67,91) | 31(12,19) | $\chi^{2}$=230.282 | 0.012673 |
| No | 55(31,24) | 286(151,135) |  |  |
| **Burping** |  |  |  |  |
| Yes | 131(48,83) | 26(14,12) | $\chi^{2}$=267.331 | 0.108857 |
| No | 40(24,16) | 333(175,158) |  |  |
| **Flatus** |  |  |  |  |
| Yes | 111(43,68) | 12(6,6) | $\chi^{2}$=258.524 | 1.0273e-7 |
| No | 55(26,29) | 352(186,166) |  |  |
| **Loose Stools** |  |  |  |  |
| Yes | 84(31,53) | 7(5,2) | $\chi^{2}$=294.349 | 0.000002 |
| No | 39(20,19) | 400(205,195) |  |  |
| **Hard Stools** |  |  |  |  |
| Yes | 90(34,56) | 14(4,10) | $\chi^{2}$=353.514 | 0.720100 |
| No | 17(9,8) | 409(214,195) |  |  |
| **Constipation** |  |  |  |  |
| Yes | 102(39,63) | 18(5,13) | $\chi^{2}$=306.426 | 0.183925 |
| No | 28(12,16) | 382(205,177) |  |  |
| **Diarrhea** |  |  |  |  |
| Yes | 92(37,55) | 13(6,7) | $\chi^{2}$=215.626 | 5.0449e-9 |
| No | 63(32,31) | 362(187,175) |  |  |
| **Tenesmus** |  |  |  |  |
| Yes | 129(53,74) | 12(6,6) | $\chi^{2}$=346.400 | 0.011508 |
| No | 29(12,17) | 360(190,170) |  |  |
| **Urgency** |  |  |  |  |
| Yes | 73(26,47) | 3(1,2) | $\chi^{2}$=358.703 | 0.000049 |
| No | 24(12,12) | 430(222,208) |  |  |

^a^ The analysis excluded individuals who were not infected with COVID-19, and a paired chi-square test was conducted to examine the correlation between variables, with a significance level set at *P*<0.05 indicating statistical significance.

^b^ The results indicated that, apart from hunger pain, burping, hard stools, the incidence rates of all other gastrointestinal symptoms following COVID-19 infection were correlated with gender.

**Table 5 Correlation Between 15 Gastrointestinal Symptoms and Age (Before Infection)**

|  | **Age(years)** | | **Statistic** | ***P*** |
| --- | --- | --- | --- | --- |
|  | **No** | **Yes** |  |  |
| **Abdominal pain** | 14.91±1.889 | 15.70±1.890 | t=-4.041 | <0.001 |
| **Acid reflux** | 14.97±1.901 | 15.78±1.828 | t=-3.513 | <0.001 |
| **Heartburn** | 15.00±1.910 | 16.07±1.629 | t=-4.143 | <0.001 |
| **Hunger pain** | 14.91±1.923 | 15.61±1.778 | t=-3.786 | <0.001 |
| **Nausea** | 14.99±1.895 | 15.68±1.910 | t=-2.972 | 0.003 |
| **Bloated** | 14.85±1.883 | 15.95±1.755 | t=-6.078 | <0.001 |
| **Borborygmus** | 14.88±1.913 | 15.48±1.847 | t=-3.571 | <0.001 |
| **Burping** | 14.87±1.895 | 15.64±1.839 | t=-4.417 | <0.001 |
| **Flatus** | 14.95±2.203 | 15.77±1.863 | t=-3.794 | <0.001 |
| **Loose Stools** | 14.97±2.156 | 15.96±1.889 | t=-4.129 | <0.001 |
| **Hard Stools** | 14.99±2.185 | 15.77±1.834 | t=-3.479 | <0.001 |
| **Constipation** | 14.88±1.875 | 15.84±1.856 | t=-4.998 | <0.001 |
| **Diarrhea** | 14.90±1.876 | 15.87±1.864 | t=-4.798 | <0.001 |
| **Tenesmus** | 14.82±1.886 | 15.88±1.758 | t=-5.929 | <0.001 |
| **Urgency** | 15.02±1.894 | 15.49±1.967 | t=-2.046 | 0.041 |

^a^ The independent samples t-test was employed to assess the correlation between gastrointestinal symptoms and age, with a significance level set at *P*<0.05 indicating statistical significance.

^b^ The results indicated that all gastrointestinal symptoms before COVID-19 infection were correlated with age.

**Table 6 Correlation Between Gastrointestinal Symptoms and Gender (Acute infection)**

|  | **Acute infection** | | **Statistic** | ***P*** |
| --- | --- | --- | --- | --- |
|  | **Yes(Male, Female)** | **No(Male, Female)** |  |  |
| **Before infection** |  |  |  |  |
| **Yes** | 307(142,165) | 18(12,6) | $\chi^{2}$=193.517 | 1.0115e-10 |
| **No** | 81 (37,44) | 124(70,54) |  |  |

^a^ Stratified paired chi-square tests were conducted on the surveyed population infected with COVID-19, stratified by gender, with a significance level set at *P*<0.05 indicating statistical significance.

^b^ The results indicated significant differences in the occurrence rates of gastrointestinal symptoms before and after infection for both males and females.

**Table 7 Correlation Between Gastrointestinal Symptoms and Age (Acute infection)**

|  | **Acute infection** | | **Statistic** | ***P*** |
| --- | --- | --- | --- | --- |
|  | **Yes** | **No** |  |  |
| **Before infection** |  |  |  |  |
| **Yes** | 15.51±2.312 | 14.89±1.779 | $F=$5.917 | 0.000566 |
| **No** | 14.51±1.833 | 14.85±1.969 |  |  |

^a^ A one-way ANOVA test was employed to analyze age differences among different population groups, with a significance level set at *P*<0.05 indicating statistical significance.

^b^ The results indicated that there were age differences between individuals who exhibited gastrointestinal symptoms both before and after COVID-19 infection, those who only showed symptoms after infection, and those who did not display any gastrointestinal symptoms at all.

**Table 8 Correlation Between Gastrointestinal Symptoms and Educational level (Acute infection)**

|  | **Acute infection** | | **Statistic** | ***P*** |
| --- | --- | --- | --- | --- |
|  | **Yes** | **No** |  |  |
| **Before infection** |  |  |  |  |
| **6^th^ grade** |  |  |  |  |
| Yes | 25 | 2 | $\chi^{2}$=12.317 | 0.002350 |
| No | 15 | 15 |  |  |
| **7^th^ grade** |  |  |  |  |
| Yes | 53 | 5 | $\chi^{2}$=37.941 | 0.000546 |
| No | 24 | 40 |  |  |
| **8^th^ grade** |  |  |  |  |
| Yes | 55 | 3 | $\chi^{2}$=47.194 | 2.5605e-12 |
| No | 12 | 29 |  |  |
| **9^th^ grade** |  |  |  |  |
| Yes | 27 | 3 | $\chi^{2}$=13.224 | 0.145996 |
| No | 9 | 12 |  |  |
| **10^th^ grade** |  |  |  |  |
| Yes | 42 | 3 | $\chi^{2}$=16.364 | 0.343750 |
| No | 7 | 8 |  |  |
| **11^th^ grade** |  |  |  |  |
| Yes | 60 | 1 | $\chi^{2}$=27.699 | 0.011719 |
| No | 10 | 9 |  |  |
| **12^th^ grade** |  |  |  |  |
| Yes | 43 | 1 | $\chi^{2}$=30.616 | 0.375000 |
| No | 4 | 11 |  |  |

^a^ When stratifying by educational levels and conducting a paired chi-squared test on gastrointestinal symptoms among COVID-19-infected individuals, with a significance level set at *P*<0.05 indicating statistical significance.

^b^ The results revealed significant differences in the occurrence rates of gastrointestinal symptoms before and after infection among 6^th^ graders, 7^th^ graders, 8^th^ graders, and 11^th^ graders.

**Table 9 Correlation Between Gastrointestinal Symptoms and Gastrointestinal History (Acute infection)**

|  | **Acute infection** | | **Statistic** | ***P*** |
| --- | --- | --- | --- | --- |
|  | **Yes** | **No** |  |  |
| **Before infection** |  |  |  |  |
| **Yes** | 307(234,73) | 18(15,3) | $\chi^{2}$=193.517 | 1.0115e-10 |
| **No** | 81 (76,5) | 124(118,6) |  |  |

^a^ Stratifying by gastrointestinal disease history, a paired chi-squared test was conducted to examine gastrointestinal symptoms among individuals infected with COVID-19, with a significance level set at *P*<0.05 indicating statistical significance.

^b^ The results revealed a significant difference in the occurrence rates of gastrointestinal symptoms before and after infection among students with a history of gastrointestinal disease.

**Table 10 Correlation Between Sleep Quality and Gender (Acute infection)**

|  | **Acute infection** | | | | **Statistic** | ***P*** |
| --- | --- | --- | --- | --- | --- | --- |
|  | **Very good** | **Fairly good** | **Fairly bad** | **Very bad** |  |  |
| **Before infection** |  |  |  |  |  |  |
| **Very good** | 304(159,145) | 63(34,29) | 9(6,3) | 8(2,6) | $\chi^{2}$=254.408 | 3.4782e-7 |
| **Fairly good** | 22(7,15) | 76(36,40) | 13(8,5) | 4(2,2) |  |  |
| **Fairly bad** | 2(0,2) | 4(2,2) | 11(3,8) | 4(1,3) |  |  |
| **Very bad** | 0(0,0) | 1(1,0) | 1(0,1) | 1(0,1) |  |  |

^a^ Stratified by gender, a paired chi-squared test was conducted to assess the sleep quality of individuals infected with COVID-19, with a significance level set at *P*<0.05 indicating statistical significance.

^b^ The results indicated that both males and females experienced significant changes in sleep quality before and after infection. The study showed that female infected individuals exhibited lower sleep quality in comparison.

**Table 11 Correlation Between Sleep Quality and Educational Level (Acute infection)**

|  | **Before infection** | | | | | | | | | | | | | | | | | | | | | | | | | | | |
| --- | --- | --- | --- | --- | --- | --- | --- | --- | --- | --- | --- | --- | --- | --- | --- | --- | --- | --- | --- | --- | --- | --- | --- | --- | --- | --- | --- | --- |
|  | **6^th^ grade** | | | | **7^th^ grade** | | | | **8^th^ grade** | | | | **9^th^ grade** | | | | **10^th^ grade** | | | | **11^th^ grade** | | | | **12^th^ grade** | | | |
|  | **Very good** | **Fairly good** | **Fairly bad** | **Very bad** | **Very good** | **Fairly good** | **Fairly bad** | **Very bad** | **Very good** | **Fairly good** | **Fairly bad** | **Very bad** | **Very good** | **Fairly good** | **Fairly bad** | **Very bad** | **Very good** | **Fairly good** | **Fairly bad** | **Very bad** | **Very good** | **Fairly good** | **Fairly bad** | **Very bad** | **Very good** | **Fairly good** | **Fairly bad** | **Very bad** |
| **Acute infection** |  |  |  |  |  |  |  |  |  |  |  |  |  |  |  |  |  |  |  |  |  |  |  |  |  |  |  |  |
| **Very good** | 39 | 3 | 0 | 0 | 86 | 2 | 0 | 0 | 59 | 2 | 0 | 0 | 29 | 1 | 0 | 0 | 34 | 2 | 0 | 0 | 30 | 6 | 1 | 0 | 26 | 5 | 1 | 0 |
| **Fairly good** | 5 | 4 | 0 | 0 | 15 | 9 | 0 | 0 | 17 | 14 | 1 | 0 | 8 | 8 | 0 | 0 | 3 | 10 | 1 | 0 | 11 | 16 | 2 | 1 | 4 | 15 | 0 | 0 |
| **Fairly bad** | 2 | 0 | 2 | 1 | 3 | 3 | 0 | 0 | 2 | 0 | 1 | 0 | 2 | 0 | 0 | 0 | 0 | 5 | 2 | 0 | 0 | 4 | 4 | 0 | 0 | 1 | 2 | 0 |
| **Very bad** | 1 | 0 | 0 | 0 | 1 | 0 | 1 | 0 | 3 | 0 | 0 | 0 | 0 | 0 | 2 | 0 | 1 | 0 | 0 | 0 | 1 | 2 | 1 | 0 | 1 | 2 | 0 | 1 |
| **Statistic** | $\chi^{2}$=255.699 | | | | | | | | | | | | | | | | | | | | | | | | | | | |
| ***P*** | 1.9962e-7 | | | | | | | | | | | | | | | | | | | | | | | | | | | |

^a^ Stratified by educational levels, a paired chi-squared test was conducted to assess the sleep quality of individuals infected with COVID-19, with a significance level set at *P*<0.05 indicating statistical significance.

^b^ Overall, the surveyed population showed differences in sleep quality before and after infection.

**Table 12 Incidence of Gastrointestinal Symptoms at Different Follow-up Time Points**

|  | **Gastrointestinal Symptom** | | | | | | | | | |
| --- | --- | --- | --- | --- | --- | --- | --- | --- | --- | --- |
|  | **1^st^ mon post-infection** | | **3^rd^ mon post-infection** | | **6^th^ mon post-infection** | | **1^st^ mon VS 6^th^ mon** | | **Acute infection VS 6^th^ mon** | |
|  | **Yes** | **No** | **Yes** | **No** | **Yes** | **No** | **Yes** | **No** | **Yes** | **No** |
| **Gender** |  |  |  |  |  |  |  |  |  |  |
| **Male** |  |  |  |  |  |  |  |  |  |  |
| Yes | 142 | 37 | 133 | 15 | 131 | 8 | 126 | 22 | 123 | 56 |
| No | 6 | 76 | 6 | 107 | 3 | 119 | 8 | 105 | 11 | 71 |
| **Female** |  |  |  |  |  |  |  |  |  |  |
| Yes | 168 | 41 | 157 | 16 | 159 | 3 | 158 | 15 | 155 | 40 |
| No | 5 | 55 | 5 | 91 | 6 | 101 | 7 | 89 | 24 | 50 |
| ***P*** | 1.3674e-13 | | 0.002887 | | 0.823803 | | 0.003185 | | 9.3953e-8 | |
| **Age(years)** |  |  |  |  |  |  |  |  |  |  |
| Yes | 15.45±2.293 | 14.71±2.010 | 15.49±2.318 | 15.00±1.871 | 15.47±2.306 | 15.10±2.183 | 15.48±2.329 | 15.17±1.871 | 15.46±2.343 | 14.89±1.965 |
| No | 15.45±2.067 | 14.80±1.927 | 14.33±1.225 | 14.79±1.981 | 13.11±1.616 | 14.89±1.947 | 13.71±1.383 | 14.85±1.970 | 14.57±1.886 | 14.91±1.951 |
| ***P*** | 0.007485 | | 0.003433 | | 0.000695 | | 0.001069 | | 0.019768 | |
| **Educational level** |  |  |  |  |  |  |  |  |  |  |
| **6^th^ grade** |  |  |  |  |  |  |  |  |  |  |
| Yes | 25 | 15 | 23 | 3 | 23 | 1 | 23 | 3 | 24 | 16 |
| No | 1 | 16 | 1 | 30 | 4 | 29 | 4 | 27 | 3 | 14 |
| **7^th^ grade** |  |  |  |  |  |  |  |  |  |  |
| Yes | 57 | 20 | 52 | 7 | 52 | 3 | 51 | 8 | 49 | 18 |
| No | 2 | 43 | 3 | 60 | 4 | 63 | 5 | 58 | 7 | 38 |
| **8^th^ grade** |  |  |  |  |  |  |  |  |  |  |
| Yes | 57 | 10 | 52 | 7 | 54 | 1 | 51 | 8 | 51 | 16 |
| No | 2 | 30 | 3 | 37 | 0 | 44 | 3 | 37 | 3 | 29 |
| **9^th^ grade** |  |  |  |  |  |  |  |  |  |  |
| Yes | 25 | 11 | 21 | 5 | 23 | 0 | 21 | 5 | 20 | 16 |
| No | 1 | 14 | 2 | 23 | 0 | 28 | 2 | 23 | 3 | 12 |
| **10^th^ grade** |  |  |  |  |  |  |  |  |  |  |
| Yes | 42 | 7 | 40 | 4 | 39 | 2 | 39 | 5 | 37 | 12 |
| No | 2 | 9 | 1 | 15 | 0 | 19 | 0 | 16 | 2 | 9 |
| **11^th^ grade** |  |  |  |  |  |  |  |  |  |  |
| Yes | 60 | 10 | 59 | 2 | 57 | 3 | 57 | 4 | 57 | 13 |
| No | 1 | 9 | 1 | 18 | 1 | 19 | 1 | 18 | 1 | 9 |
| **12^th^ grade** |  |  |  |  |  |  |  |  |  |  |
| Yes | 42 | 5 | 42 | 2 | 41 | 1 | 41 | 3 | 39 | 8 |
| No | 2 | 10 | 0 | 15 | 0 | 17 | 0 | 15 | 2 | 10 |
| ***P*** | 1.3674e-13 | | 0.004324 | | 0.168638 | | 0.004601 | | 2.6618e-13 | |
| **Gastrointestinal history** |  |  |  |  |  |  |  |  |  |  |
| **Yes** |  |  |  |  |  |  |  |  |  |  |
| Yes | 71 | 7 | 69 | 2 | 71 | 0 | 69 | 2 | 69 | 9 |
| No | 0 | 9 | 2 | 14 | 1 | 15 | 3 | 13 | 3 | 6 |
| **No** |  |  |  |  |  |  |  |  |  |  |
| Yes | 239 | 71 | 221 | 29 | 219 | 11 | 215 | 35 | 209 | 101 |
| No | 11 | 122 | 9 | 184 | 8 | 205 | 12 | 181 | 18 | 115 |
| ***P*** | 3.4133e-55 | | 9.9212e-50 | | 8.5034e-50 | | 8.5034e-50 | | 8.5034e-50 | |

^a^ Conducting follow-up assessments at 1st month, 3rd month, and 6th month after COVID-19 infection, we stratified the data by gender, educational level, and gastrointestinal disease history. Paired chi-squared tests and rank-sum tests were used for the analysis of the correlation between variables.

^b^ The results indicated that when stratified by gender, males exhibited a significant improvement in gastrointestinal symptoms compared to females in the early stages of follow-up.

^c^ When stratified by educational level, there was a notable improvement in gastrointestinal symptoms among sixth-grade and middle school students.

^d^ When stratified by gastrointestinal disease history, the results showed that individuals with a history of gastrointestinal diseases experienced a significant improvement in gastrointestinal symptoms throughout the entire follow-up period compared to those without a history of gastrointestinal diseases.

**Table 13 Sleep Quality Assessment at Different Follow-up Time Points**

|  | **Sleep Quality** | | | | | | | | | | | | | | | | | | | |
| --- | --- | --- | --- | --- | --- | --- | --- | --- | --- | --- | --- | --- | --- | --- | --- | --- | --- | --- | --- | --- |
|  | **1^st^ mon post-infection** | | | | **3^rd^ mon post-infection** | | | | **6^th^ mon post-infection** | | | | **1^st^ mon VS 6^th^ mon** | | | | **Acute infection VS 6^th^ mon** | | | |
|  | **Very good** | **Fairly good** | **Fairly bad** | **Very bad** | **Very good** | **Fairly good** | **Fairly bad** | **Very bad** | **Very good** | **Fairly good** | **Fairly bad** | **Very bad** | **Very good** | **Fairly good** | **Fairly bad** | **Very bad** | **Very good** | **Fairly good** | **Fairly bad** | **Very bad** |
| **Gender** |  |  |  |  |  |  |  |  |  |  |  |  |  |  |  |  |  |  |  |  |
| **Male** |  |  |  |  |  |  |  |  |  |  |  |  |  |  |  |  |  |  |  |  |
| Very good | 155 | 11 | 0 | 0 | 179 | 8 | 0 | 0 | 186 | 3 | 0 | 0 | 178 | 9 | 0 | 0 | 152 | 13 | 0 | 1 |
| Fairly good | 29 | 37 | 5 | 2 | 8 | 57 | 1 | 0 | 5 | 52 | 0 | 0 | 11 | 44 | 0 | 0 | 32 | 35 | 4 | 2 |
| Fairly bad | 3 | 7 | 7 | 0 | 2 | 2 | 12 | 0 | 0 | 6 | 6 | 1 | 2 | 8 | 6 | 0 | 6 | 9 | 2 | 0 |
| Very bad | 0 | 1 | 4 | 0 | 0 | 0 | 0 | 2 | 0 | 0 | 0 | 2 | 0 | 0 | 0 | 3 | 1 | 4 | 0 | 0 |
| **Female** |  |  |  |  |  |  |  |  |  |  |  |  |  |  |  |  |  |  |  |  |
| Very good | 134 | 25 | 3 | 0 | 157 | 5 | 0 | 0 | 164 | 4 | 0 | 0 | 153 | 8 | 1 | 0 | 133 | 23 | 5 | 0 |
| Fairly good | 22 | 43 | 5 | 1 | 10 | 65 | 2 | 0 | 9 | 63 | 3 | 0 | 18 | 56 | 3 | 0 | 30 | 37 | 3 | 1 |
| Fairly bad | 1 | 6 | 9 | 1 | 1 | 5 | 17 | 0 | 0 | 2 | 16 | 1 | 2 | 5 | 15 | 0 | 3 | 5 | 7 | 1 |
| Very bad | 3 | 1 | 6 | 2 | 0 | 0 | 0 | 4 | 0 | 0 | 1 | 3 | 0 | 0 | 1 | 4 | 3 | 3 | 5 | 2 |
| ***P*** | 0.033372 | | | | 0.144342 | | | | 0.176289 | | | | 0.016059 | | | | 0.011574 | | | |
| **Educational level** |  |  |  |  |  |  |  |  |  |  |  |  |  |  |  |  |  |  |  |  |
| **6^th^ grade** |  |  |  |  |  |  |  |  |  |  |  |  |  |  |  |  |  |  |  |  |
| Very good | 36 | 6 | 0 | 0 | 39 | 1 | 0 | 0 | 42 | 1 | 0 | 0 | 39 | 1 | 0 | 0 | 39 | 3 | 0 | 0 |
| Fairly good | 3 | 6 | 0 | 0 | 4 | 10 | 0 | 0 | 1 | 10 | 0 | 0 | 4 | 10 | 0 | 0 | 3 | 6 | 0 | 0 |
| Fairly bad | 1 | 1 | 2 | 1 | 0 | 0 | 2 | 0 | 0 | 0 | 2 | 0 | 0 | 0 | 2 | 0 | 1 | 1 | 2 | 1 |
| Very bad | 0 | 1 | 0 | 0 | 0 | 0 | 0 | 1 | 0 | 0 | 0 | 1 | 0 | 0 | 0 | 1 | 0 | 1 | 0 | 0 |
| **7^th^ grade** |  |  |  |  |  |  |  |  |  |  |  |  |  |  |  |  |  |  |  |  |
| Very good | 84 | 4 | 0 | 0 | 97 | 3 | 0 | 0 | 100 | 0 | 0 | 0 | 97 | 3 | 0 | 0 | 84 | 4 | 0 | 0 |
| Fairly good | 12 | 12 | 0 | 0 | 3 | 15 | 0 | 0 | 0 | 19 | 0 | 0 | 3 | 15 | 0 | 0 | 12 | 12 | 0 | 0 |
| Fairly bad | 2 | 2 | 2 | 0 | 0 | 1 | 2 | 0 | 0 | 1 | 1 | 0 | 0 | 2 | 1 | 0 | 2 | 4 | 0 | 0 |
| Very bad | 1 | 0 | 1 | 0 | 0 | 0 | 0 | 0 | 0 | 0 | 0 | 0 | 0 | 0 | 0 | 0 | 1 | 0 | 1 | 0 |
| **8^th^ grade** |  |  |  |  |  |  |  |  |  |  |  |  |  |  |  |  |  |  |  |  |
| Very good | 56 | 5 | 0 | 0 | 66 | 2 | 0 | 0 | 68 | 2 | 0 | 0 | 65 | 3 | 0 | 0 | 53 | 7 | 0 | 0 |
| Fairly good | 12 | 16 | 4 | 0 | 3 | 19 | 0 | 0 | 5 | 19 | 0 | 0 | 6 | 16 | 0 | 0 | 16 | 15 | 1 | 0 |
| Fairly bad | 0 | 1 | 2 | 0 | 1 | 3 | 4 | 0 | 0 | 2 | 2 | 0 | 2 | 4 | 2 | 0 | 2 | 0 | 1 | 0 |
| Very bad | 1 | 0 | 2 | 0 | 0 | 0 | 0 | 0 | 0 | 0 | 0 | 0 | 0 | 0 | 0 | 0 | 2 | 1 | 0 | 0 |
| **9^th^ grade** |  |  |  |  |  |  |  |  |  |  |  |  |  |  |  |  |  |  |  |  |
| Very good | 30 | 0 | 0 | 0 | 37 | 2 | 0 | 0 | 38 | 0 | 0 | 0 | 37 | 2 | 0 | 0 | 28 | 2 | 0 | 0 |
| Fairly good | 8 | 8 | 0 | 0 | 1 | 8 | 0 | 0 | 1 | 9 | 0 | 0 | 2 | 7 | 0 | 0 | 10 | 6 | 0 | 0 |
| Fairly bad | 1 | 1 | 0 | 0 | 0 | 0 | 2 | 0 | 0 | 1 | 1 | 0 | 0 | 1 | 1 | 0 | 1 | 1 | 0 | 0 |
| Very bad | 0 | 0 | 2 | 0 | 0 | 0 | 0 | 0 | 0 | 0 | 0 | 0 | 0 | 0 | 0 | 0 | 0 | 1 | 1 | 0 |
| **10^th^ grade** |  |  |  |  |  |  |  |  |  |  |  |  |  |  |  |  |  |  |  |  |
| Very good | 29 | 6 | 1 | 0 | 29 | 3 | 0 | 0 | 30 | 2 | 0 | 0 | 27 | 4 | 1 | 0 | 27 | 7 | 1 | 1 |
| Fairly good | 2 | 10 | 0 | 2 | 1 | 18 | 2 | 0 | 3 | 17 | 1 | 0 | 4 | 16 | 0 | 1 | 2 | 10 | 1 | 1 |
| Fairly bad | 0 | 4 | 3 | 0 | 2 | 0 | 3 | 0 | 0 | 2 | 2 | 1 | 2 | 1 | 2 | 0 | 2 | 4 | 1 | 0 |
| Very bad | 0 | 0 | 1 | 0 | 0 | 0 | 0 | 2 | 0 | 0 | 1 | 1 | 0 | 0 | 1 | 1 | 0 | 0 | 1 | 0 |
| **11^th^ grade** |  |  |  |  |  |  |  |  |  |  |  |  |  |  |  |  |  |  |  |  |
| Very good | 29 | 7 | 1 | 0 | 39 | 2 | 0 | 0 | 40 | 1 | 0 | 0 | 38 | 3 | 0 | 0 | 28 | 8 | 1 | 0 |
| Fairly good | 11 | 13 | 5 | 1 | 2 | 22 | 0 | 0 | 2 | 23 | 1 | 0 | 4 | 19 | 1 | 0 | 12 | 12 | 4 | 2 |
| Fairly bad | 0 | 3 | 5 | 0 | 0 | 2 | 11 | 0 | 0 | 1 | 9 | 1 | 0 | 3 | 9 | 1 | 1 | 3 | 4 | 0 |
| Very bad | 0 | 1 | 2 | 1 | 0 | 0 | 0 | 2 | 0 | 0 | 0 | 2 | 0 | 0 | 0 | 2 | 0 | 2 | 1 | 1 |
| **12^th^ grade** |  |  |  |  |  |  |  |  |  |  |  |  |  |  |  |  |  |  |  |  |
| Very good | 24 | 7 | 1 | 0 | 28 | 0 | 0 | 0 | 32 | 0 | 0 | 0 | 28 | 0 | 0 | 0 | 26 | 3 | 3 | 0 |
| Fairly good | 3 | 15 | 1 | 0 | 4 | 19 | 1 | 0 | 2 | 17 | 1 | 0 | 6 | 16 | 2 | 0 | 7 | 11 | 1 | 0 |
| Fairly bad | 0 | 1 | 2 | 0 | 0 | 1 | 5 | 0 | 0 | 1 | 5 | 0 | 0 | 2 | 4 | 0 | 0 | 1 | 2 | 0 |
| Very bad | 1 | 0 | 2 | 1 | 0 | 0 | 0 | 1 | 0 | 0 | 0 | 1 | 0 | 0 | 0 | 1 | 1 | 2 | 0 | 1 |
| ***P*** | 0.028814 | | | | 0.144342 | | | | 0.121437 | | | | 0.025271 | | | | 0.009878 | | | |
| **Gastrointestinal history** |  |  |  |  |  |  |  |  |  |  |  |  |  |  |  |  |  |  |  |  |
| **Yes** |  |  |  |  |  |  |  |  |  |  |  |  |  |  |  |  |  |  |  |  |
| Very good | 35 | 6 | 0 | 0 | 42 | 4 | 0 | 0 | 43 | 1 | 0 | 0 | 41 | 4 | 1 | 0 | 32 | 7 | 1 | 1 |
| Fairly good | 11 | 13 | 4 | 1 | 2 | 20 | 1 | 0 | 3 | 22 | 2 | 0 | 5 | 16 | 1 | 1 | 13 | 11 | 4 | 1 |
| Fairly bad | 0 | 3 | 4 | 0 | 0 | 3 | 12 | 0 | 0 | 2 | 9 | 2 | 0 | 5 | 9 | 1 | 1 | 2 | 4 | 0 |
| Very bad | 0 | 0 | 7 | 1 | 0 | 0 | 0 | 2 | 0 | 0 | 1 | 1 | 0 | 0 | 1 | 1 | 0 | 4 | 3 | 1 |
| **No** |  |  |  |  |  |  |  |  |  |  |  |  |  |  |  |  |  |  |  |  |
| Very good | 254 | 30 | 3 | 0 | 294 | 9 | 0 | 0 | 307 | 6 | 0 | 0 | 290 | 13 | 0 | 0 | 253 | 29 | 4 | 0 |
| Fairly good | 40 | 67 | 6 | 2 | 16 | 92 | 2 | 0 | 11 | 93 | 1 | 0 | 24 | 84 | 2 | 0 | 49 | 61 | 3 | 2 |
| Fairly bad | 4 | 10 | 12 | 1 | 3 | 4 | 17 | 0 | 0 | 6 | 13 | 0 | 4 | 8 | 12 | 0 | 8 | 12 | 6 | 1 |
| Very bad | 3 | 2 | 3 | 1 | 0 | 0 | 0 | 4 | 0 | 0 | 0 | 4 | 0 | 0 | 0 | 4 | 4 | 3 | 1 | 1 |
| ***P*** | 0.033372 | | | | 0.144342 | | | | 0.176289 | | | | 0.032397 | | | | 0.016232 | | | |

^a^ Conducting follow-up assessments at 1st month, 3rd month, and 6th month after COVID-19 infection, we stratified the data by gender, educational level, and gastrointestinal disease history. Paired chi-squared tests were employed for analysis.

^b^ The results revealed that when stratified by gender, males demonstrated a significant improvement in sleep quality compared to females in the early stages of follow-up. By the end of the follow-up period, it was still the case that sleep quality significantly improved for males compared to the acute infection period and early follow-up.

^c^ When stratified by educational level, in the first month following COVID-19 infection, there was a noticeable improvement in the sleep quality of middle school students.

^d^ When stratified by gastrointestinal disease history, individuals with no history of gastrointestinal diseases showed a significant improvement in sleep quality compared to those with a history of gastrointestinal diseases.
